# The GNAT: A new tool for processing NMR data

**DOI:** 10.1002/mrc.4717

**Published:** 2018-03-25

**Authors:** Laura Castañar, Guilherme Dal Poggetto, Adam A. Colbourne, Gareth A. Morris, Mathias Nilsson

**Affiliations:** ^1^ School of Chemistry University of Manchester Oxford Road Manchester M13 9PL UK

**Keywords:** analysis, NMR, diffusion, DOSY, MATLAB, multiway, processing, relaxation, software, toolbox

## Abstract

The GNAT (General NMR Analysis Toolbox) is a free and open‐source software package for processing, visualising, and analysing NMR data. It supersedes the popular DOSY Toolbox, which has a narrower focus on diffusion NMR. Data import of most common formats from the major NMR platforms is supported, as well as a GNAT generic format. Key basic processing of NMR data (e.g., Fourier transformation, baseline correction, and phasing) is catered for within the program, as well as more advanced techniques (e.g., reference deconvolution and pure shift FID reconstruction). Analysis tools include DOSY and SCORE for diffusion data, ROSY T_1_/T_2_ estimation for relaxation data, and PARAFAC for multilinear analysis. The GNAT is written for the MATLAB® language and comes with a user‐friendly graphical user interface. The standard version is intended to run with a MATLAB installation, but completely free‐standing compiled versions for Windows, Mac, and Linux are also freely available.

## 1 INTRODUCTION

A variety of software packages are available for processing high‐resolution NMR data. They can be classified into three main categories: (a) supplied by spectrometer manufacturers,^1^, ^2^, ^3^, ^4^, ^5^ (b) commercial packages,^6^, ^7^, ^8^, ^9^, ^10^, ^11^ and (c) free software.^12^, ^13^, ^14^, ^15^, ^16^ In recent years, an increasing number of free software packages, often produced by individual researchers or groups to cover specific needs, have been produced.^17^, ^18^, ^19^, ^20^, ^21^, ^22^, ^23^, ^24^, ^25^ One example is the popular DOSY (Diffusion‐Ordered SpectroscopY) Toolbox,^23^ a free and open‐source software package typically intended for processing NMR diffusion (a.k.a. DOSY^26^, ^27^) data, although sometimes used for other purposes. The first official version was released in 2009, and it has since become a useful tool for many scientists, complementing other NMR software.

Here, we describe the GNAT (General NMR Analysis Toolbox), a free and open‐source platform, released under the General Public License, for processing and analysing NMR data. This new toolbox is based on and replaces the DOSY Toolbox (although existing versions of the DOSY Toolbox will remain available). The intention is to provide a more general tool for analysing NMR data, independent of acquisition platform, to complement the manufacturers' software offerings. As open‐source software, it allows users to implement their own algorithms. The GNAT works within the MATLAB® environment, which has extensive libraries of mathematical computation and visualisation routines, making the GNAT flexible and easily extendable. The aim of this publication is to present the major features of the GNAT program to the wider NMR community and to give an overview of its general features. This publication is intended in part as a short introductory manual, highlighting the most important features and illustrating them with real experimental data. The GNAT can be downloaded from our website (http://nmr.chemistry.manchester.ac.uk/), where we intend to publish updates as well as making more detailed and updated documentation and test data available. All the example data used in this publication can also be found at the DOI: http://dx.doi.org/10.17632/pyr9688wvb.1.

## 2 FEATURES

The GNAT is intended to provide a user‐friendly tool for analysing NMR data, primarily high resolution, independent of source. As such, it currently supports import of data from Bruker, Varian, and JEOL instruments, but it can easily be extended to support other formats. Many of the analysis tools in GNAT are tailored for use with series of NMR spectra, whether as a function of gradient level (typical for diffusion data), of an incremented delay (typical for relaxation data), or of some other variable, such as spectra from a time‐series. Array structures as a function of up to two independent variables are supported (e.g., spectra as a function of both diffusion and relaxation). Data import therefore supports the standard Varian array structure and Bruker “ser” files, as well as a series of consecutively numbered 1D datasets in either format. Import of processed 1D spectra is also supported for Bruker data. All of these options are easily accessible from the “File” menu. Data can subsequently be saved in the internal GNAT file format, either in binary or in human‐readable ASCII format. Whole data structures can also be saved in standard MATLAB format as *.mat, and read back separately into MATLAB using the “load” command. Data can be saved as raw unprocessed data, or in the form of “FIDs” (Free Induction Decays) produced by inverse Fourier transform of the complex or real processed spectra. The latter option irreversibly saves any processing, such as baseline correction or reference deconvolution, in the form of a FID that can then of course be reopened and processed further. Access to some important processing parameters is available from the “Edit” menu.

**Figure 1:**
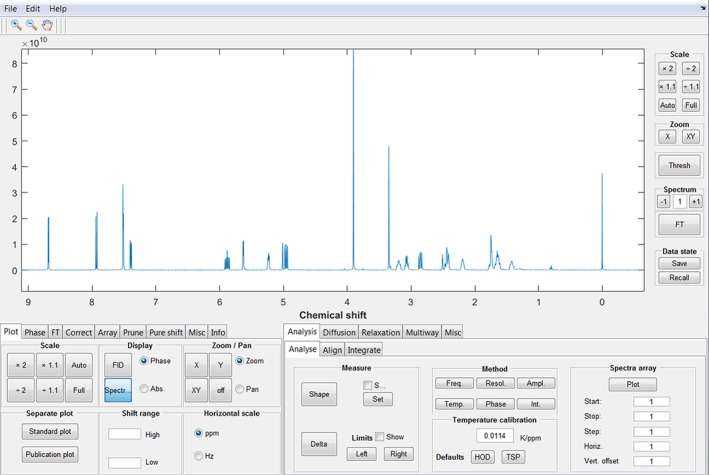
Main window of the graphical user interface of the GNAT. Most of the processing and analysis functionalities are easily accessible from here. The spectrum shown is the conventional 500 MHz ^1^H NMR spectrum of quinine

The graphical user interface (GUI) consists of a main window (Figure 1), displaying spectra or FIDs, from which access to most processing and analysis features is available. When more advanced functions are used, for example, diffusion or relaxation 2D plots (DOSY and ROSY [Relaxation‐Ordered SpectroscopY], respectively), separate windows are opened, in which method‐specific features are available. The GUI is divided between basic processing and features in the left tab group (e.g., Fourier transformation and plot control) and more advanced analysis (e.g., DOSY and PARAFAC [PARAllel FACtor]) in the right tab group. Table 1 lists the most important processing and analysis features implemented in the current version of the GNAT.

**Table 1:** List of processing and analysis functions implemented in the GNAT

Processing	Basic	Fourier transformation	
		Zero‐filling	
		Apodisation	
		Referencing	
		Phase correction^28^	
		Baseline correction^29^	
	Advanced	Reference deconvolution^30^	
		Pure shift interferogram construction^31^, ^32^, ^33^, ^34^	
Analysis	Basic	Peak width	
		Frequency shift	
		Resolution change	
		Amplitude change	
		Temperature change	
		Phase change^35^	
		Integral change	
	Advanced	Univariate methods	DOSY^26^, ^27^, ^36^
			ROSY^37^, ^38^, ^39^
		Multivariate methods	SCORE/RSCORE^40^, ^41^
			OUTSCORE^42^
			LOCODOSY^43^
			ILT^44^, ^45^
			DECRA^46^, ^47^, ^48^, ^49^
			FDM/RRT^50^, ^51^, ^52^
			ICA^53^, ^54^, ^55^
			MCR^56^, ^57^, ^58^
			PARAFAC^59^, ^60^, ^61^, ^62^, ^63^, ^64^, ^65^
			Slicing^66^

## 3 PROCESSING AND ANALYSING NMR DATA

The GNAT software is mainly designed for analysing series of spectra such as diffusion and relaxation data, but also caters for processing of single FIDs. In order to optimise the information extractable from spectra, a variety of tools are included. Many of these are standard for most NMR processing software, and only a short description will be included here.

### 3.1 Data processing (left tab group)

The “Plot” tab contains the controls for display; these includes setting of spectral limits and scale units (ppm or Hz). Separate plots can be produced for inclusion in reports and publications.

The “Phase” tab contains the controls for phase correction. Manual correction is performed by setting a pivot point and varying the zeroth and first order phases. The automatic phase correction algorithm is based on the simple and effective early work in this field.^28^, ^67^ Phase correction can be performed either for a whole array of spectra simultaneously, or for individual spectra, by switching between “Global” and “Individual” mode.

**Figure 2:**
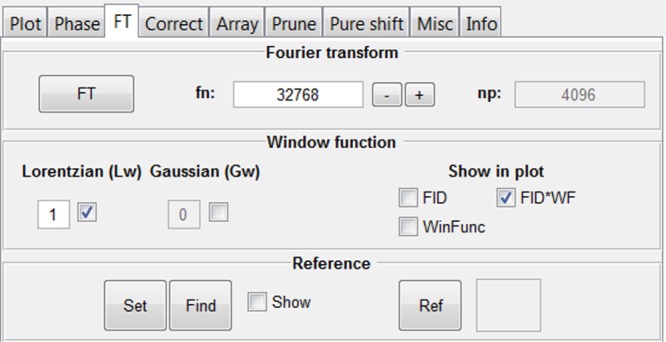
Fourier transformation (FT) tab. Zero‐filling, apodisation, and spectrum referencing functions are available here

**Figure 3:**
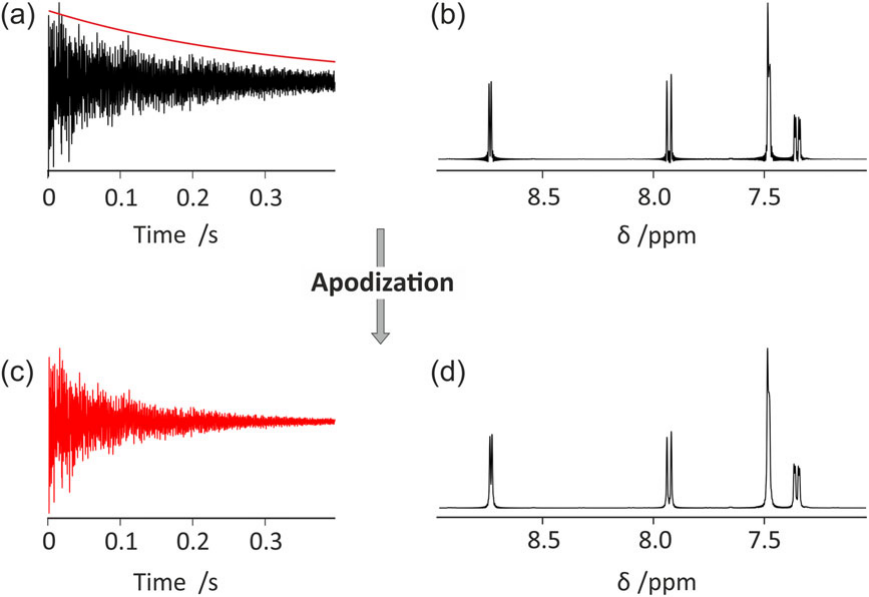
Processing a truncated FID with the GNAT. (a) Simultaneous display of the truncated FID and (red line) window function, (b) spectrum resulting from Fourier transformation with no weighting, (c) weighted FID, and (d) final spectrum after Fourier transformation of the weighted FID (c)

The “FT” tab implements Fourier transformation of FIDs (Figure 2). The number of data points in the spectrum is determined by the Fourier number (*fn*), the total number of complex data points, to allow for zero‐filling or truncation to arbitrary size. Window functions are used to multiply the FID, typically to enhance sensitivity or resolution in the resultant spectrum and/or to suppress so‐called sinc wiggles where FID data are truncated. Figure 3 shows an example where a line‐broadening window function is applied in order to enhance sensitivity and suppress sinc wiggles. The interface allows display of both the window function and the weighted FID. In the GNAT, window functions are implemented with Lorentzian and Gaussian parameters by multiplying the FID by the function below, where *t* is time within the FID and *lw* and *gw* are the additional Lorentzian and Gaussian linewidths at half height, respectively.

$$e^{-t\;\pi \;\mathrm{lw}-{\left(\frac{\pi \;\mathrm{gw}\;t}{2\;\sqrt{\ln2)}}\right)}^{2}\;}$$

Thus, using *gw* = 0 convolutes the raw spectrum with a Lorentzian line of width *lw* Hz, whereas using *lw* = 0 convolutes the raw spectrum with a Gaussian line of width *gw* Hz.

Using a negative value for *lw* and positive for *gw* allows resolution enhancement by Lorentz–Gauss transformation, whereas using both values positive gives a Voigt lineshape. In the same tab, the user can also set a chemical shift reference.

**Figure 4:**
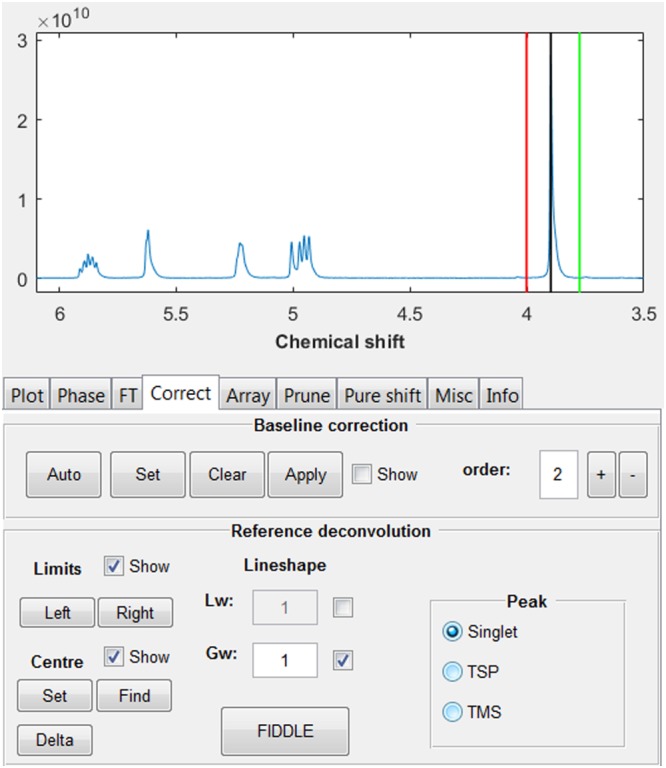
“Correct” tab. Baseline correction and reference deconvolution processing functions are available here

The “Correct” tab implements various forms of correction of the data (Figure 4). Manual baseline correction is achieved by selecting the regions of the spectrum that contain signal; the remaining baseline is then fitted to a polynomial of user‐defined order and subtracted from the spectrum. The automatic baseline correction^29^ iteratively adjusts the definitions of the baseline regions to achieve the best baseline correction for a specified order of polynomial.

A very powerful, but still underused, tool for correcting systematic errors is reference deconvolution.^30^ This works by using the differences between an ideal reference signal and a resolved experimental signal to correct for systematic errors (e.g., lineshape distortions, phase errors, and frequency shifts) in the whole spectrum. In the GNAT, the reference signal can be a simple singlet (for spectra of any nucleus), or tetramethylsilane (TMS ) or TSP‐*d*_4_ (trimethylsilylpropanoate) for ^1^H spectra. In the latter two cases, the known ^29^Si and ^13^C satellite patterns of the reference signals are included in the calculation. Reference deconvolution is particularly valuable for multivariate analysis methods, because these rely heavily on the linearity of the data (which requires that the spectral shapes for individual resonances remain the same in all increments of a dataset). In our laboratory, reference deconvolution is used routinely, and we and others have seen significant improvements in many investigations.^41^, ^49^, ^62^, ^63^, ^68^, ^69^, ^70^, ^71^, ^72^, ^73^, ^74^, ^75^, ^76^ The implementation of reference deconvolution processing in the GUI is shown in Figure 4. The user simply uses two cursors to define the spectral limits of the reference peak, typically the whole peak plus a small piece of baseline either side, the type of reference signal, and the target lineshape (the shape required for the experimental reference peak after correction, as specified by *lw* and *gw* in the weighting function above). The correction is executed by pressing the FIDDLE (Free Induction Decay Deconvolution for Lineshape Enhancement) button.

**Figure 5:**
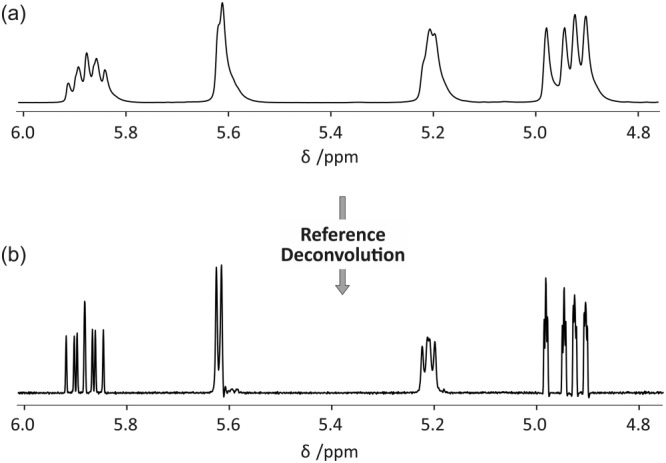
Schematic illustration of simple reference deconvolution processing using data acquired with non‐optimal shimming. Deconvoluting the raw experimental spectrum (a) with the methoxy lineshape contained between the red and green lines in Figure 4 and reconvoluting with a 1 Hz Gaussian wide lineshape give the corrected spectrum (b) with clean lineshapes

The method is demonstrated here (Figure 5) on data from a proton experiment on a quinine sample in which the homogeneity of the static field was deliberately perturbed by poor shimming. The quinine methoxy group at 3.9 ppm (Figure 4) was used as reference, with a 1 Hz Gaussian target lineshape to correct the severe lineshape distortions in the rest of the spectrum (Figure 5).

The “Array” tab contains an interface for plotting spectra from an array; see Section 3.2.1 below.

The “Prune” tab contains an interface to remove (prune) part of the data before further analysis. Individual array elements and/or spectral regions can be removed as desired.

**Figure 6:**
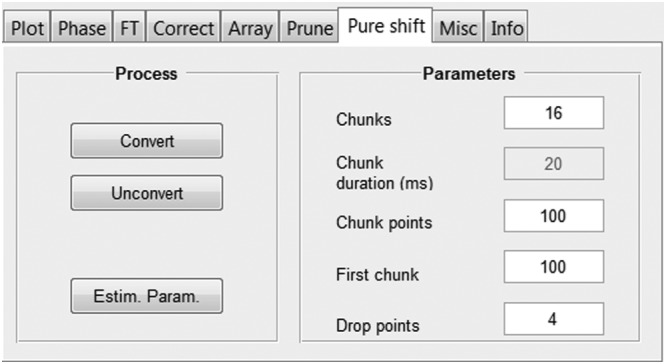
“Pure shift” tab for performing pure shift FID reconstruction

**Figure 7:**
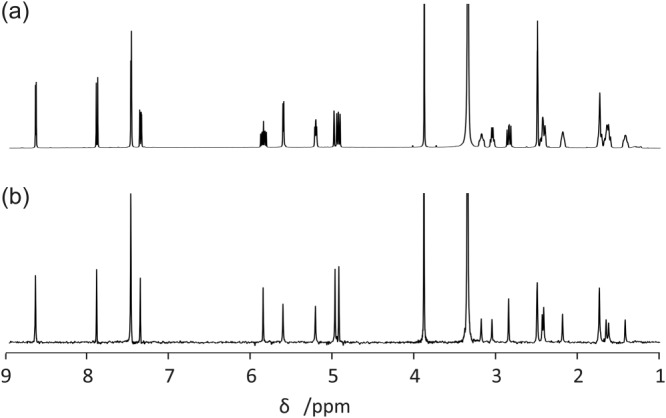
(a) Conventional and (b) interferogram pure shift ^1^H NMR spectra of quinine, processed with the GNAT software

The “Pure shift” tab (Figure 6) contains an interface for constructing a pure shift FID from an interferogram experiment (including pure shift DOSY^77^, ^78^, ^79^). Pure shift methods,^31^, ^32^, ^33^, ^34^ where the resolution of NMR spectra is improved by suppressing the multiplet structure caused by homonuclear couplings (Figure 7), can be classified into two main groups on the basis of the way the data are acquired: pseudo‐2D (interferogram)^80^, ^81^, ^82^, ^83^ or real‐time.^84^, ^85^, ^86^, ^87^, ^88^ Real‐time experiments directly generate a single FID that after standard Fourier transformation gives a homodecoupled 1D NMR spectrum. In interferogram experiments, a synthetic 1D homodecoupled FID (or “interferogram”) is constructed by concatenating data chunks extracted from individual time‐domain datasets acquired in 2D mode. The GNAT includes the post‐processing needed for interferogram experiments to reconstruct the 1D pure shift FID; the parameters required are read in with the raw data but can also be adjusted by the user if necessary. “Chunks” refers to the number of increments in the dataset; “Chunk duration” the duration of a single FID chunk; “Chunk points” the number of data points per chunk; “First chunk” the number of data points in the first chunk; and “Drop points” the number of data points to be discarded from the start of each chunk.

The “Misc” tab currently provides buttons to save and recall the state of the current data (these functions can also be found in the shortcuts in the top left corner of the main GUI) and an interface to listen to your FIDs (for the musically inclined).

The “Info” tab displays information about the data, for example, import information and error messages. A log file (GNATlog.txt in the current working directory) keeps a running copy of the GNAT information displayed in this tab. It can be saved separately, for example, for efficient identification and reporting of bugs.

### 3.2 Data analysis (right tab group)

In the data analysis section of the interface, various methods have been implemented for specific data analyses. The first level of the tab group is divided into different categories of data: (general) Analysis, Diffusion, Relaxation, Multiway, and Misc; each in turn contains a range of methods. The analysis functions typically use the spectral region displayed in the main window of the GUI (active spectral window). Further regions can be excluded from analysis in the “Prune tab” (see above).

#### 3.2.1 The “Analysis” tab group

Here, the user can find some general analysis tools, which are mainly intended for analysis of a series of spectra.

**Figure 8:**
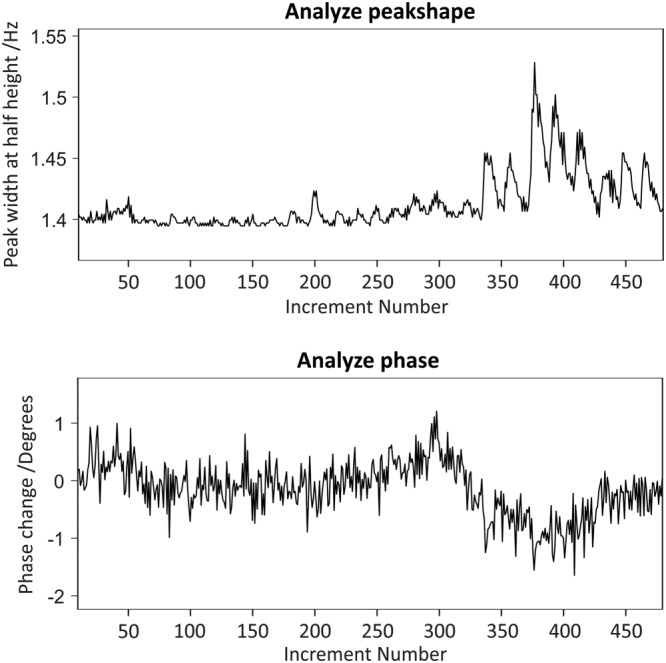
Testing the stability of a newly installed Bruker Neo 500 MHz NMR spectrometer. 480 ^1^H NMR experiments were acquired over 8 hr using a sample of acetone in D_2_O. The GNAT analysis tool is used to monitor changes in the linewidth (top) and phase (bottom) of the water signal as a function of time. The effects of room temperature variation are apparent

The “Analyse” tab contains an interface for the analysis of individual and arrayed experiments (see Figure 1). The user can make simple measurements of linewidth (“Shape” function) and frequency difference (“Delta” function). Using “Spectra array,” the user can choose how to plot an array, by choosing start, stop, and step points and vertical and horizontal offsets. These settings are also used to determine the array elements to use in the different analysis methods in this tab. “Freq” plots the change in frequency (for the highest peak in the display window that is above a user‐set threshold) as a function of spectrum number; “Resol” the peak width at half height; “Ampl” the peak amplitude; “Temp” the temperature estimated from the peak position (vide infra); “Phase” the absolute signal phase deviation from pure absorption mode (the actual calculation uses the dispersion mode; see below); and “Int” the integral (for a defined integral region). Typical outputs are shown below (Figure 8) for resolution and phase, showing the stability of one of our spectrometers over an 8‐hr period.

The change in phase is determined by comparing the peak excursions of a near‐dispersion mode signal.^35^ This relies on having a (close to) Lorentzian lineshape, which can be achieved in practice by applying a Lorentzian window function that is broad compared with the experimental linewidth, and phasing the first spectrum in a series approximately to absorption mode. The algorithm then temporarily shifts the phase by 90° and uses the positive and negative peak excursions to calculate the phase deviation.

**Figure 9:**
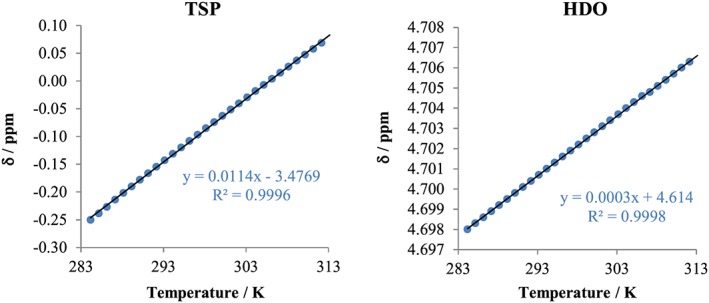
Dependence of the apparent chemical shifts of HDO and TSP signals on temperature, as reported by Topspin using standard lock‐based referencing. ^1^H NMR spectra were acquired between 11 °C and 40 °C (in 1 °C steps)

The estimated change in temperature with spectrum number assumes that the sample is dissolved in D_2_O and that the dominant peak is either of residual water (HDO) or of a resonance with a negligible temperature coefficient of chemical shift. In the former case, the temperature dependence is that of the primary isotope effect of hydrogen in D_2_O; in the latter, it is dominated by the (relatively large) temperature sensitivity of the deuterium chemical shift of the D_2_O lock material. The calibration used is based on experimental measurements of the temperature dependences of the HDO and TSP chemical shifts, as reported by Topspin software, for a standard Bruker test sample of HDO and TSP in D_2_O. This yielded temperature coefficients of 2.97 × 10^−4^ ppm/K for HOD and 1.14 × 10^−2^ ppm/K for TSP (Figure 9). When performing temperature analysis, which relies on the temperature dependence of the chemical shift, it is important to ensure that the TSP/HOD peak remains within the active spectral window for all spectra of the array.

The “Align” tab contains an interface to allow a series of spectra to be manually aligned in frequency space.

The “Integrate” tab contains an interface to allow integration of spectral peaks, including correction of baseline offset and tilt. Integral regions can be picked manually or automatically and normalised if desired. Integral values and/or regions can be exported in a human‐readable ASCII format, and regions can be reimported from an export file.

#### 3.2.2 The “Diffusion” tab group

**Figure 10:**
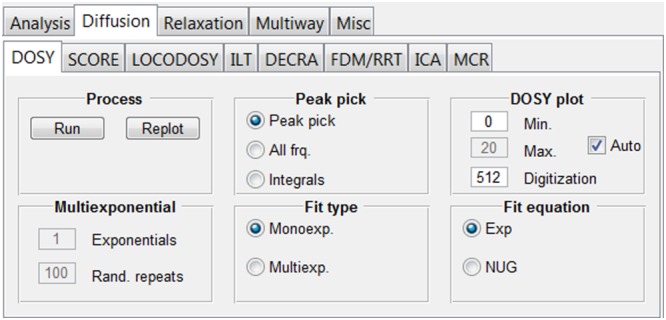
“Diffusion” tab group. From here, it is possible to access tabs where the functionalities of univariate (DOSY and ILT) and multivariate (SCORE, LOCODOSY, DECRA, FDM/RRT, ICA, and MCR) methods are implemented

Here, the user can find various options for analysing diffusion NMR data (Figure 10). The general description of this topic is beyond the scope of this publication, and the reader is referred, as a starting point, to selected review and application articles.^26^, ^27^, ^89^, ^90^, ^91^, ^92^, ^93^, ^94^, ^95^, ^96^, ^97^ Many of the methods in this and other tabs are highly sensitive to the quality of the input data, because of the assumptions on which the algorithms rely. It is therefore prudent to take extra care in the preparation of data, with, for example, careful baseline correction and phasing. We find it particularly helpful to use reference deconvolution, where a suitable reference signal is available, to correct for the many systematic errors present in experimental data. The GNAT has some automatic settings, for example, from parameters imported with the raw data, but user‐defined parameters and parameter adjustments are possible using “Edit > Settings > Diffusion.” (e.g., when the diffusion data are imported but the class of pulse sequence used cannot be determined automatically, the GNAT will assume a bipolar sequence type; this can be changed here.)

The “DOSY” tab contains controls for typical DOSY^26^, ^27^, ^36^ processing. The default processing is HR‐DOSY^98^ in which each peak is assumed to originate from a single species, and therefore a monoexponential fit is performed to some version of the Stejskal–Tanner equation:^99^, ^100^

(1) $$I_{g}=I_{0}\;e^{-\gamma^{2}\delta^{2}g^{2}D\Delta^{\prime }},$$

where *I*_0_ is the signal intensity in the absence of diffusion, γ is the magnetogyric ratio of the diffusion‐encoded spins, δ is the gradient pulse duration, *g* is the gradient amplitude, *D* is the diffusion coefficient, and Δ^′^ is the corrected diffusion‐encoding time. An empirical correction of Equation (1) for the effects of spatially non‐uniform pulsed field gradients (NUG) can also be used but requires calibration of the spectrometer/probe.^101^ An attempt to fit to an arbitrary number of exponentials^102^, ^103^ (e.g., when a signal contains contributions from several components) can be made by selecting “Multiexp” and choosing the number of exponentials. The fitting routine will reduce the number of components until statistically significant results are obtained. Any fit can be performed using either automated peak picking, all points in the spectrum, or pre‐set integral regions.

**Figure 11:**
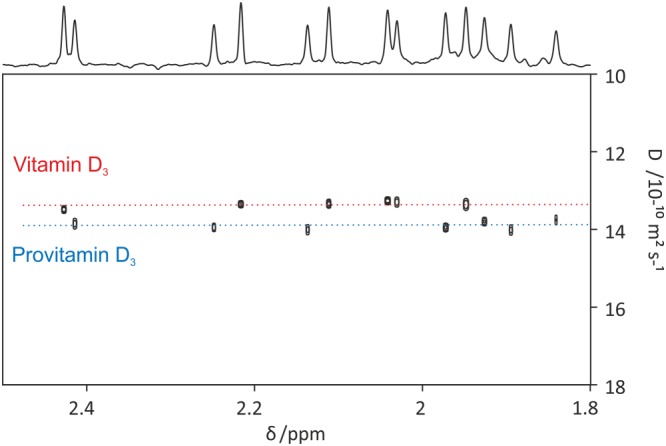
PSYCHE‐iDOSY spectrum of a mixture containing 50 mM each of vitamin D_3_ and provitamin D in acetone‐*d*_6_. Data for the original publication^79^ were processed with the GNAT software. Prior to DOSY analysis, 1D data were processed by pure shift reconstruction, zero‐filling, apodisation (with 1 Hz additional Gaussian linewidth), Fourier transformation, and phase and baseline correction. The DOSY plot was constructed using peak picking with a user‐defined threshold and a monoexponential fit

The resulting DOSY spectrum is displayed in a separate DOSY GUI with individual controls for plotting and analysing the data. These include plots of fits and residuals, projections, and separate plots intended for reports. A separate text file with the relevant fit statistics is also accessible via the DOSY GUI. Figure 11 shows an example of a pure shift DOSY spectrum obtained with the GNAT for a mixture of provitamin and vitamin D_3_ in DMSO‐*d*_6_, using monoexponential fitting.^79^

**Figure 12:**
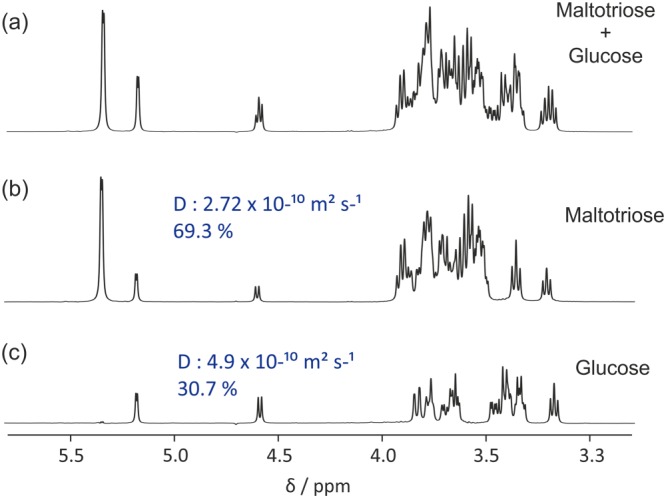
(a) Conventional ^1^H NMR spectrum of an equimolar mixture of maltotriose, glucose, and TSP‐*d*_4_ in D_2_O, and (b) and (c) component spectra corresponding to maltotriose and glucose, respectively, obtained by SCORE processing of a diffusion‐encoded data set. The percentages shown are the fractions of the total signal integral present for each component spectrum. Prior to analysis, data were processed with zero‐filling, phase and baseline correction, Fourier transformation, and reference deconvolution using TSP‐*d*_4_ signal as reference with a 2 Hz Gaussian target lineshape. SCORE analysis was performed using two fitted components and non‐negativity constraint

The “SCORE” tab contains the controls for SCORE (Speedy COmponent REsolution) analysis. SCORE^40^, ^41^, ^104^, ^105^ is a type of multivariate processing in which entire component spectra are produced by a successful analysis. The OUTSCORE (Optimized Unmixing of True Spectra for COmponent REsolution)^42^ variant, in which the separation criterion maximises spectral differences rather than minimising residuals, as in SCORE, is also available. The user chooses the number of components to fit and has the options to use a pure exponential (Equation (1)) or a NUG‐corrected^101^ decay and to do the fit with or without non‐negativity constraints (i.e., with only positive values of the spectra and diffusion decays allowed). The result is presented as fitted spectra and diffusion decays together with an estimate of the relative signal integrals. In Figure 12, the fitted SCORE components from a mixture of maltotriose and glucose are shown, together with the mixture spectrum. As an option, diagnostic plots of residuals, leverages, and residuals versus leverages can be plotted. These plots, inspired by the N‐way toolbox,^106^ are useful for identifying spectral regions and/or gradient levels that are behaving in an unexpected way and could therefore usefully be excluded from the fitting, or for indicating that the number of components chosen was incorrect.

The “LOCODOSY” tab contains the controls for LOCODOSY (LOcal COvariance Diffusion‐Ordered SpectroscopY) fitting. LOCODOSY^43^ is a hybrid between univariate (e.g., DOSY) and multivariate (e.g., SCORE) data processing. It takes advantage of the enhanced statistical leverage of multivariate methods while seeking to alleviate one of their disadvantages, that typically only a small number of components can be resolved by such methods. The spectrum is divided into individual segments that are each assumed to contain signals from only a small number of components. For each region, the maximum number of components is specified (either manually or automatically), and a multivariate fit is performed. A full DOSY spectrum is then assembled from the individual segments (for more detail, see the original publication^43^). Automated analysis is performed by pressing the “Auto” button in the “Segment” part and pressing “Run.” LOCODOSY can provide very good results but is strongly dependent on high quality data and appropriate choice of segmentation. The current options for the multivariate method to use are SCORE, OUTSCORE, and DECRA (Direct Exponential Curve Resolution Algorithm; see below).

The “ILT” tab contains controls for an implementation of what is commonly known as the inverse Laplace transform (ILT).^44^ The decay of signal with gradient amplitude in a diffusion NMR experiment contains information on the complete distribution of compounds of different sizes, which in principle is accessible via the ILT. In practice, however, this is an ill‐posed problem with an infinite number of solutions. There are a number of methods available for diffusion NMR that constrain the mathematical problem in different ways to allow unique solutions to be found.^36^, ^45^, ^72^, ^107^, ^108^, ^109^, ^110^, ^111^, ^112^, ^113^, ^114^, ^115^, ^116^, ^117^, ^118^, ^119^ They all have different advantages and disadvantages and require careful interpretation, but this is beyond the scope of this publication. (In principle, the HR‐DOSY approach is a very strict, but useful, constraint on the ILT, although it is not normally described as such in the context of diffusion NMR data). The current implementation of the ILT in the GNAT is based on the MATLAB Regularisation Tools,^120^, ^121^ as described by Day.^114^ The default values chosen should give a sensible result in many cases, but the most important parameters are all under user control.

The “DECRA” tab contains the controls for DECRA fitting. DECRA^46^, ^47^, ^48^, ^49^ is a very fast multivariate processing method that exploits the fact that diffusion NMR data ideally show pure exponential behaviour with increasing gradient amplitude squared. The only user input is the number of components to be fitted.

The “FDM/RRT” tab contains controls for the FDM (Filter Diagonalization Method)^50^ and RRT (Regularized Resolvent Transform)^51^ for analysing diffusion NMR data.^52^ This method performs a type of ILT (as defined above) and displays the results in a DOSY plot.

The “ICA” tab contains the controls for ICA (Independent Component Analysis)^54^ processing of diffusion NMR data. ICA is a multivariate method that separates components based on assumptions about their statistical independence and the non‐Gaussian behaviour of signals. It can be very effective for diffusion NMR analysis of mixtures where there is little spectral overlap.^55^ In the GNAT, this processing is implemented using the fast‐ICA algorithm.^53^

The “MCR” tab contains the controls for MCR (Multivariate Curve Resolution)^56^, ^58^ analysis of diffusion NMR data. Results here are highly dependent on starting guesses, and are primarily useful for refining fitted solutions subject to sensible constraints. In the GNAT implementation, the starting guesses are implemented using PCA‐VARIMAX^57^ and DECRA.^46^ These starting points can then be refined by imposing a non‐negativity constraint and/or by forcing the decay to be either a pure exponential or of a shape determined by a NUG calibration.^101^

#### 3.2.3 The “Relaxation” tab group

**Figure 13:**
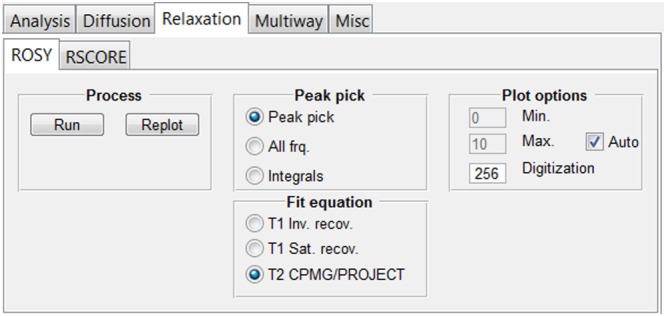
“Relaxation” tab. From here, it is possible to access other sub‐tabs in which the functionalities of univariate (ROSY) and multivariate (RSCORE) methods are implemented

Here, the user can find various ways to analyse relaxation data (Figure 13), a common and important part of NMR.^122^ The general description of this topic is beyond the scope of this publication, and the reader is referred, as a starting point, to selected books.^123^, ^124^ The GNAT imports relaxation data in standard format from Varian or Bruker, but user‐defined parameters and changes are possible using “Edit > Settings > Relaxation.” For Bruker data, delay values are imported from a *vclist* or *vdlist* file.

**Figure 14:**
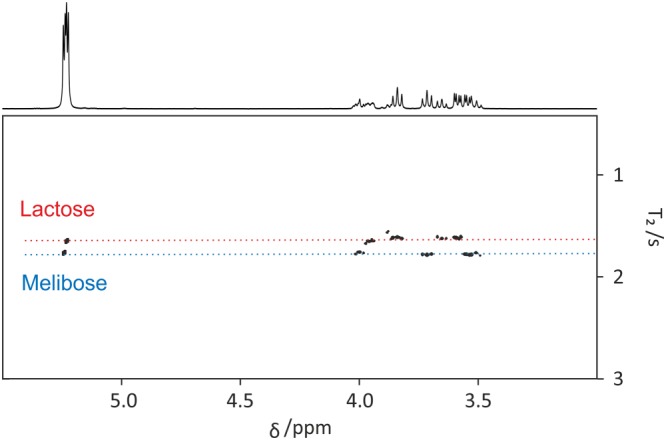
REST_2_ ROSY spectrum of a mixture of lactose, melibiose, and TSP‐*d*_4_ in D_2_O. Data from the original publication^39^ were processed with the GNAT software. Prior to ROSY analysis, data were processed with zero‐filling, Fourier transformation, phase and baseline correction, and reference deconvolution using TSP‐*d*_4_ signal as reference with a 2 Hz Gaussian target lineshape. The ROSY plot was constructed using peak picking with a user‐defined threshold and a *T*_2_ CPMG/PROJECT fit. The “Loop duration” parameter (“Edit” > “Setting” > “Relaxation”) was set appropriately for the PROJECT *T*_2_ filter used

The “ROSY” tab includes controls for relaxation processing with ROSY^9^, ^38^ display, as shown in Figure 14. (Various other names for such analyses have been suggested in the literature.^37^, ^125^) A ROSY display is analogous to DOSY in that it plots relaxation time or rate versus chemical shift in a pseudo‐2D plot. As with DOSY, the user controls whether the fitting is done using automatic peak picking, for all data points, or for pre‐set integral regions (see above). There is a choice between *T*_1_ inversion recovery,^126^ *T*_1_ saturation recovery,^127^ and *T*_2_ analysis.^128^, ^129^, ^130^ The resulting ROSY spectrum is displayed in a separate ROSY GUI, with individual controls for plotting and analysing the data. These includes plots of fits and residuals, projections, and separate plots intended for reports. A separate text file with the relevant fit statistics is also accessible via the ROSY GUI. A ROSY display of REST_2_ (Relaxation‐Encoded Selective TOCSY using *T*_2_ weighting)^39^ data is shown in Figure 14.

The “RSCORE” tab includes an implementation of SCORE^41^ and OUTSCORE^42^ analysis for relaxation data. The user can choose between *T*_2_ and *T*_1_ fitting functions and whether or not to use a non‐negativity constraint.

#### 3.2.4 The “Multiway” tab group

Multiway analysis is a superset of multivariate analysis in which the data have three or more dimensions, and is of increasing interest in chemistry^131^ and NMR spectroscopy.^62^, ^63^, ^64^, ^70^, ^73^, ^74^, ^132^, ^133^, ^134^, ^135^

**Figure 15:**
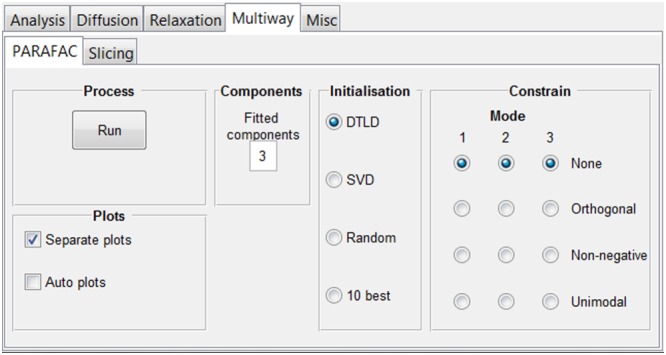
“Multiway” tab group. From here, it is possible to access tabs where PARAFAC and slicing functionalities are implemented

**Figure 16:**
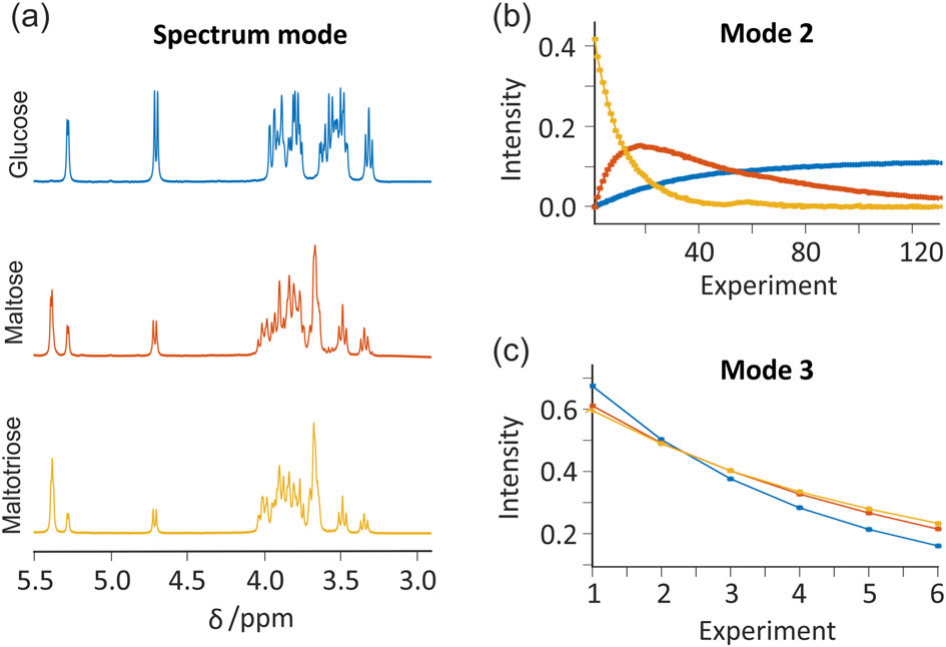
PARAFAC analysis of the hydrolysis of maltotriose. Diffusion NMR experiments were performed continuously over the course of the reaction. Separate plots for each of the independent dimensions—(a) NMR spectra, (b) concentration as a function of time, and (c) attenuation as a function of gradient amplitude—are generated. Information about each of the species present during the reaction can be easily extracted. Data from the original publication^63^ were processed with the GNAT software. Prior to analysis, data were processed using zero‐filling, Fourier transformation, phase and baseline correction, and reference deconvolution using the pivalic acid signal (at 1.233 ppm) as reference with a 2 Hz Gaussian target lineshape. PARAFAC analysis was performed using three fitted components and without constraints. Plots were obtained using the “Separate plots” option

The “PARAFAC” tab incorporates an interface to the PARAFAC functionality in the N‐way toolbox^106^ (Figure 15). The PARAFAC model assumes that the data dimensions (or modes) are mathematically independent (i.e., the data are multilinear).^59^, ^60^, ^61^, ^64^ When this assumption holds, PARAFAC typically yields results for each mode that are directly physically relevant, for example, corresponding to individual chemical components rather than to arbitrary linear combinations as in many other forms of multivariate analysis. One example is when diffusion NMR data are acquired during the course of a chemical reaction.^63^, ^70^ The resultant data then vary with the three independent dimensions of Larmor frequency, gradient amplitude (causing signal decay as a result of diffusion), and time (causing signal amplitude changes as a result, e.g., of chemical reaction) for the species involved. Figure 16 shows the result of a PARAFAC analysis of the hydrolysis of maltotriose.^63^ Such PARAFAC analyses can be very powerful, but they are very sensitive to deviations from linearity such as changes in the NMR spectrum due to variation in, for example, shimming or temperature. In some cases, these variations can be corrected with reference deconvolution.^30^, ^75^ In the interface, the user can choose to constrain the algorithm (e.g., by non‐negativity), to try different initialisation methods, and to produce a series of diagnostic plots.

The “Slicing” tab contains an implementation of PowerSlicing for further analysis with the PARAFAC algorithm.^61^, ^106^ PowerSlicing^66^ and Slicing^132^ are directly related to DECRA^46^ in that they take advantage of exponential behaviour, for example, in some diffusion or relaxation NMR data, to produce an artificially trilinear system. PowerSlicing also shares a disadvantage of DECRA in that it fails when data deviate from pure exponential behaviour.^49^ By analysing data with the PARAFAC algorithm rather than just with DECRA, the user has the possibility to incorporate constraints in order to improve results.

#### 3.2.5 The “Misc” tab group

Here, we have implemented some tools that do not easily fit in the other categories but may well still be useful.

The “Sim DOSY” tab contains controls for simulating DOSY data, for further analysis in the GNAT or for export. Typical parameters for a pulsed field gradient NMR experiment are available, and the user can decide, for example, the diffusion coefficients and *T*_2_ values of the individual simulated peaks in the spectrum.

The “Bin” tab incudes a facility to bin NMR spectra for further analysis, for example, by PCA (principal components analysis). The binned data can be exported in MATLAB (*.mat) or *.csv formats.

The “ICOSHIFT” tab incorporates an interface to the ICOSHIFT (interval correlation optimized shifting) algorithm^136^ for peak alignment, for example, in metabolomics data.

## 4 CONCLUSION

The GNAT is a free and open‐source tool for the analysis of NMR data. It aspires to be a platform for easy and fast implementation of useful processing methods. Revised versions will be downloadable from our website (http://nmr.chemistry.manchester.ac.uk/); our intention is to continuously support the software with new implementations, improvements, and bug fixes.

### 4.1 Experimental section

Experimental spectra were recorded on three different spectrometers: 500 MHz Bruker Avance II+, 500 MHz Bruker Avance Neo, and 400 MHz Varian INOVA. Eight different samples were used: sample 1 contained 100 mM quinine and TMS in DMSO‐*d*_6_; sample 2 contained 50 mM quinine in DMSO‐*d*_6_; sample 3 contained 1% vol/vol acetone in D_2_O; sample 4 contained TSP in D_2_O; sample 5 contained an equimolar mixture (50 mM) of vitamin D_3_ and provitamin D_3_ in acetone‐*d*_6_ (more information in original publication^79^); sample 6 contained an equimolar mixture (100 mM) of glucose and maltotriose in D_2_O; sample 7 contained an equimolar mixture (100 mM) of lactose and melibose in D_2_O (more information in original publication^39^); and sample 8 initially contained 18 mM maltotriose, 25 mM pivalic acid (as a reference), and 90 mM sulphuric acid in D_2_O (more information in original publication^63^).
